# Dosimetric potential of knowledge‐based planning model trained with HyperArc plans for brain metastases

**DOI:** 10.1002/acm2.13836

**Published:** 2022-11-05

**Authors:** Tomohiro Sagawa, Yoshihiro Ueda, Haruhi Tsuru, Tatsuya Kamima, Shingo Ohira, Mikoto Tamura, Masayoshi Miyazaki, Hajime Monzen, Koji Konishi

**Affiliations:** ^1^ Department of Radiation Oncology Osaka International Cancer Institute Osaka Japan; ^2^ Department of Medical Physics and Engineering Graduate School of Medicine Osaka University Suita Japan; ^3^ Radiation Oncology Department Cancer Institute Hospital Japanese Foundation for Cancer Research Tokyo Japan; ^4^ Department of Medical Physics Graduate School of Medical Sciences Kindai University Sayama Japan

**Keywords:** brain metastases, HyperArc, knowledge‐based planning, RapidPlan, treatment planning

## Abstract

**Objective:**

Dosimetric potential of knowledge‐based RapidPlan planning model trained with HyperArc plans (Model‐HA) for brain metastases has not been reported. We developed a Model‐HA and compared its performance with that of clinical volumetric modulated arc therapy (VMAT) plans.

**Methods:**

From 67 clinical stereotactic radiosurgery (SRS) HyperArc plans for brain metastases, 47 plans were used to build and train a Model‐HA. The other 20 clinical HyperArc plans were recalculated in RapidPlan system with Model‐HA. The model performance was validated with the 20 plans by comparing dosimetric parameters for normal brain tissue between clinical plans and model‐generated plans. The 20 clinical conventional VMAT‐based SRS or stereotactic radiotherapy plans (CL‐VMAT) were reoptimized with Model‐HA (RP) and HyperArc system (HA), respectively. The dosimetric parameters were compared among three plans (CL‐VMAT vs. RP vs. HA) in terms of planning target volume (PTV), normal brain excluding PTVs (Brain − PTV), brainstem, chiasm, and both optic nerves.

**Results:**

In model validation, the optimization performance of Model‐HA was comparable to that of HyperArc system. In comparison to CL‐VMAT, there were no significant differences among three plans with respect to PTV coverage (*p* > 0.17) and maximum dose for brainstem, chiasm, and optic nerves (*p* > 0.40). RP provided significantly lower *V*_20 Gy_, *V*_12 Gy_, and *V*_4 Gy_ for Brain − PTV than CL‐VMAT (*p* < 0.01).

**Conclusion:**

The Model‐HA has the potential to significantly reduce the normal brain dose of the original VMAT plans for brain metastases.

## 1 INTRODUCTION

Stereotactic radiosurgery (SRS) is a suitable treatment technique to deliver the optimal dose distribution for brain metastasis radiotherapy.^1^, ^2^ Several studies have demonstrated that SRS can deliver less radiation to normal brain tissue and better preserve cognitive function than conventional solutions.^3^, ^4^, ^5^ Volumetric modulated arc therapy (VMAT)‐based SRS with a single isocenter allows delivering doses to multiple targets simultaneously while minimizing the dose to surrounding normal tissues.^6^, ^7^, ^8^, ^9^, ^10^ In particular, newer VMAT‐based SRS solutions with a single isocenter, HyperArc (Varian Medical Systems, Palo Alto, CA), can provide a steeper dose gradient for targets with a lower workload than conventional VMAT‐based SRS.^11^

Knowledge‐based planning (KBP) models can predict the range of achievable dose–volume histograms (DVH) of organs at risk (OAR) based on library data of exiting plans. Some studies have demonstrated that KBP is effective at improving dose sparing of OARs.^12^, ^13^, ^14^, ^15^, ^16^, ^17^, ^18^, ^19^, ^20^ Several studies applying KBP to SRS for multiple brain metastases have also been reported.^19^, ^21^ However, the sparing performance of KBP model depends on the quality of library plans included in the model.^22^

Herein, we hypothesized that a KBP model with comparable optimization potential to that of the HyperArc system can be generated by training with HyperArc plans. The model could provide greater dose reduction of normal brain tissue than original manual plans, owing to the superior sparing potential of HyperArc.^11^, ^23^ Because RapidPlan models can be shared among facilities, if a model can be created with HyperArc‐like dose reduction potential, it may be possible to improve plan quality at facilities that do not have a license to use HyperArc.

The novelty and aim of this study were to develop a RapidPlan model trained with HyperArc plans (Model‐HA) and evaluate its dosimetric and mechanical performance for brain metastases. Plan parameters of Model‐HA were compared with those of HyperArc plans to confirm model performance and with those of conventional VMAT plans to assess plan quality improvement.

*Contributions*. Our main contribution are as follows:

- *Model potential*: We were able to create a model trained on HyperArc. With the same beam settings as HyperArc, the model‐based plan was able to reduce the brain dose as much as HyperArc.
- *Importance of geometry*: When the beam settings were determined manually, the model‐based plan was not able to reduce the brain dose as much as HyperArc. Beam arrangement was also found to be highly dependent on dose reduction.
- *Improving without HyperArc system*: Sharing the model would allow facilities that do not have a license to use HyperArc to improve their plans.

## 2 METHODS AND MATERIALS

### 2.1 Patient selection and structures

This study was approved by the ethics committee of the Osaka International Cancer Institute. All patients provided written informed consent. For this study, we selected 87 patients with 1–15 brain metastases treated in SRS or stereotactic radiotherapy between April 2017 and September 2019 at the Osaka International Cancer Institute. Table 1 lists the characteristics of the patients and their treatment planning. The median age of the patients was 66‐year old (range: 25–88). Among them, 67 plans were calculated with the HyperArc system, and the other 20 plans were calculated using the conventional VMAT planning system. A total of 47 out of the 67 HyperArc plans were used to train Model‐HA. The other 20 HyperArc plans were reoptimized in RapidPlan system with Model‐HA and used for model validation by comparing dosimetric parameters with clinical HyperArc plans. Subsequently, the 20 clinical conventional VMAT plans (CL‐VMAT) were reoptimized with Model‐HA (RP) and HyperArc system (HA). The dosimetric parameters were compared among three types of plans (CL‐VMAT vs. RP vs. HA) to examine the clinical application of the Model‐HA to CL‐VMAT.

**TABLE 1:** Patient characteristics

Characteristic	Value
Patients (*n* = 87)	
**Age**	
Median	66
Range	25–88
**Sex**	
Female	31
Male	56
**Number of metastases**	
Median	2
Range	1–15
**Prescribed dose**	
18 Gy/1 fraction	1
20 Gy/1 fraction	19
20 Gy/5 fractions	1
24 Gy/1 fraction	45
25 Gy/5 fractions	1
30 Gy/3 fractions	9
30 Gy/5 fractions	3
35 Gy/5 fractions	8
**Treatment planning**	
HyperArc	67
CL‐VMAT (non‐HyperArc)	20

Abbreviation: CL‐VMAT, clinical volumetric modulated arc therapy.

The gross tumor volume (GTV) was delineated on computed tomography (CT) images, which were registered with a gadolinium‐enhanced T1‐weighted magnetic resonance imaging set. Planning target volume (PTV) incorporated a 3‐mm isotropic margin from the GTV. For multiple metastases cases, a structure named PTV_all_ was defined as a union of all PTVs involved in the treatment. The OARs included in this study were brainstem, chiasm, optic nerves, and structure named Brain − PTV as a brain excluding PTVs.

### 2.2 HyperArc planning

With HyperArc, the software automatically sets the optimal single isocenter, collimator angle, and noncoplanar settings, taking into accounts the size and positioning of the targets, to provide a conformal plan with low dose to the normal brain. The arc geometry (four arc fields: a full coplanar arc field with a 0° couch and three half‐noncoplanar arc fields with a 45°, 315°, and 90°, or 270° couch) was automatically determined based on the distance between each lesion.^24^ Planners can only select or deselect some arcs but cannot modify their couch angle. An SRS normal tissue objective (NTO) is automatically set for HyperArc plans. It controls the dose falloff outside the targets and dose bridging between targets. It automatically recognizes spatial arrangements of targets for which dose bridging is likely to occur and tries to prevent it from occurring at least at dose levels higher than 17% of the prescription.

CT images for treatment planning were acquired using the CT scanner (Revolution HD; GE Medical Systems, Milwaukee, WI) with an Encompass (QFix, Avondale, PA) immobilization system. The scanning parameters were a matrix size of 512 × 512, a slice thickness of 1.0 mm, and a field of view of 350 mm.

For HyperArc planning, we used treatment planning system (TPS) Eclipse version 15.5 (Varian Inc.) with beam data from an Edge linear accelerator (Varian Inc.), which is equipped with a 2.5‐mm leaf‐width multi‐leaf collimator (MLC). All treatment plans were normalized such that 95% of PTV_all_ received the prescribed dose. Photon beams of 6‐MV flattening filter‐free (FFF) at a maximum dose rate of 1400 monitor units (MU) per minute were used. Dose calculations were performed using an analytical anisotropic algorithm (AAA) with a grid size of 1.25 mm. The optimization algorithm used was Photon Optimizer version 15.6.05. All treatment plans for this study were created by medical physicists with at least 3 years of clinical experience. In addition, the clinical plans used in this study were approved by a radiation oncologist with at least 3 years of clinical experience.

### 2.3 Model building

RapidPlan provides optimization parameters based on past treatment plans in order to create an optimal plan for each patient. A DVH estimation model is created on the basis of the information, structure sets, field geometry, dose matrices, DVH, correlated locations between targets and OARs, and dose prescription, extracted from a selected set of treatment plans. A minimum of 20 plan registration is required. The registered model can be used in the treatment planning of new patients to predict DVH and to set optimization objectives in accordance with past treatment plans. A line objective is in theory defined as a continuous objective line representing the desired DVH; objectives of this type would maximize the DVH constraint strength in the whole dose range. In practice, continuous lines are represented by a discrete number of dose–volume constraint points, and in the Eclipse implementation, these are at least five equally spaced over the dose range of the DVH. The characteristics of each planning techniques were summarized and compared in Table 2.

**TABLE 2:** Summary of the characteristics of each planning techniques

	Clinical VMAT	RapidPlan	HyperArc
Collimator angle	Manual	Manual	Optimal
Arc geometry	Manual	Manual	Noncoplanar four arcs
Optimize objective	Manual	Line objective	SRS NTO

Abbreviations: NTO, normal tissue objective; SRS, stereotactic radiosurgery; VMAT, volumetric modulated arc therapy.

The model configuration in RapidPlan was described in detail by Varian Inc.^25^ When the model is trained, an internal cross‐validation is performed for the model. The training set is first divided into 10 parts. Ten models are trained, and each time a model is trained, a different tenth of the data plans are left out and used for validating the model.

Forty‐seven clinical HyperArc plans were used as training plans to create a Model‐HA in RapidPlan system. Of the 47 plans, 17 involved a single metastasis, and the others involved multiple metastases (median, 3 mets; range, 1–15 mets). The mean values (±1 SD) of brain volume were 1429.9 ± 135.8 cm^3^. The mean values (±1 SD) of the ratio of Brain − PTV volumes receiving 50% of prescribed dose to PTV were 0.23% ± 0.15%.

### 2.4 Model validation

Twenty clinical HyperArc plans were recalculated in RapidPlan system with Model‐HA. Of the 20 plans, 9 involved a single metastasis, and the others involved multiple metastases (median, 2 mets; range, 1–8 mets). The mean values (±1 SD) of brain volume were 1396.3 ± 147.7 cm^3^. The mean values (±1 SD) of the ratio of Brain − PTV volumes receiving 50% of prescribed dose to PTV were 0.29% ± 0.16%. The model‐based plans were generated based on the same prescription dose, photon energy, field geometry, and calculation algorithm as clinical HyperArc plans. In the optimization process for RapidPlan generated plans, the SRS NTO could not be used, and the line objective for Brain − PTV was used. For other OARs, the same upper objectives as in the clinical plan were added. A minimum number of objectives were also manually added to the PTV so that the DVH curve of the PTV of the model‐based plan would be the same as that of the clinical plan. For model validation, the parameters for Brain − PTV (*V*_20 Gy_–*V*_2 Gy_ at 2 Gy intervals) were compared between clinical plans and model‐based plans.

### 2.5 Conventional VMAT planning and applying the model to conventional VMAT

CT images for CL‐VMAT planning were acquired with the same scanner and scan parameters as those used in HyperArc planning with a Double Shell Positioning System (MacroMedics BV, Waddinxveen, the Netherlands). The same TPS and contouring policy were followed. Coplanar (16 plans) and noncoplanar (4 plans) CL‐VMAT were normalized such that 95% of PTV_all_ received the prescribed dose. Photon beams of 6 MV or 6‐MV FFF at a maximum dose rate of 600 or 1400 MU/min were used. The arrangement of the isocenter location, gantry angle, collimator angle, and couch rotation were manually selected by medical physicists, depending on the number, size, and location of tumors. An AAA with a grid size of 1.25 mm was used for dose calculations. SRS NTO and line objective could not be used.

Twenty CL‐VMAT were recalculated in RapidPlan system with Model‐HA (RP) and HyperArc system (HA) with the same prescription dose, photon energy, and calculation algorithm as that used with CL‐VMAT. Of the 20 cases, 8 were single metastasis cases, and the rest were multiple metastasis cases (median, 2 mets; range, 1–10 mets). The mean values (±1 SD) of brain volume were 1417.0 ± 138.7 cm^3^. In four cases, the distance between PTV and brainstem was less than 1.0 cm (minimum distance: 3 mm), and in one case, the distance to the right optic nerve was less than 1.0 cm (7.5 mm). In optimization process for RP, SRS NTO could not be used, and the line objective for Brain − PTV was performed. For HA optimization, SRS NTO was used. Additionally, we manually added a minimum number of objectives for targets, so that the DVH of the target structure for RP and HA were similar to that of CL‐VMAT.

### 2.6 Analysis

Among three plan types (CL‐VMAT vs. RP vs. HA), the dosimetric parameters for Brain − PTV, such as *V*_20 Gy_, *V*_12 Gy_, and *V*_4 Gy_ were compared. In addition, the maximum dose (*D*_max_) delivered to key OARs in brain radiotherapy (brainstem, chiasm, and optic nerves) and parameters for the PTV_all_, such as the dose that covers 98%, 80%, 50%, 20% (*D*_98%_, *D*_80%_, *D*_50%_, and *D*_20%_) and conformity index (CI) were also compared. The CI was defined by Paddick as follows:

(1) $$\mathrm{CI}=\left(\mathrm{PT}V_{\mathrm{PD}}/\mathrm{PTV}\right)\times \left(\mathrm{PT}V_{\mathrm{PD}}/V_{\mathrm{PD}}\right)$$

where PTV_PD_ was the volume of PTV covered by the prescription dose, and *V*_PD_ was the prescription isodose volume.^26^

In order to compare the physical characteristics of CL‐VMAT and RP, the aperture area size (AAS) at each control point and the total MU in each field were calculated using in‐house software created with MATLAB R2016a (MathWorks, Natick, MA). The AAS was defined according to the following equation:

(2) $$\mathrm{AAS}=\sum_{i=1}^{n}\left[\left(\left|LP_{\mathrm{Ai}}+LP_{\mathrm{Bi}}\right|\right)\times \mathrm{Leaf}\mathrm{width}\right]$$

where *n* indicates the number of paired MLC. LP_A_ and LP_B_ were position of leaves in bank A and bank B. Global gamma analysis using the electronic portal imaging device (EPID) detector, aS1200 (Varian Inc.), was also performed to investigate deliverability. The total area and matrix size of the EPID were 40 × 40 cm^2^ and 1190 × 1190 pixels, respectively. All EPID images were acquired in the integrated acquisition mode with a source‐to‐imager distance of 150 cm. Measured dose responses were compared with planned dose responses using global gamma analysis. The gamma analysis was performed with a criterion of 3%/2 mm (dose difference and distance to agreement) and a threshold at 10% using the commercial software PerFRACTION (SUN Nuclear Corporation, Melbourne, FL).

SPSS (version 24; IBM, Armonk, NY) was used for all analyses. In statistical comparisons, a paired Wilcoxon signed‐rank test was used. In the case of *p*‐values <0.05, we rejected the null hypothesis of no difference between plans.

## 3 RESULTS

### 3.1 Model validation

The differences of dosimetric parameters for Brain − PTV between clinical HyperArc plan and model‐based plan are summarized in Table 3. Regardless of the number of metastases, the absolute differences (model plan minus clinical plan) in evaluated parameters (%) in 20–8 Gy range were similar *(p* > 0.05). Within the range of 6–2 Gy, model‐based plans provided a significantly lower (*p* < 0.01) irradiated volume for all cases (mean ± SD of the difference: −0.18% ± 0.35%, −0.81% ± 1.21%, and −2.98% ± 4.74% for *V*_6 Gy_, *V*_4 Gy_, and *V*_2 Gy_), respectively.

**TABLE 3:** Absolute difference (model plan–clinical plan) of parameters (%) for Brain − planning target volume (PTV)

Parameter (%)	All cases (*n* = 20)		Single meta cases (*n* = 9)		Multiple meta cases (*n* = 11)	
	Difference	*p‐*Value	Difference	*p‐*Value	Difference	*p‐*Value
*V*_20 Gy_	−0.00 ± 0.02	0.526	−0.01 ± 0.02	0.066	0.00 ± 0.01	0.477
*V*_18 Gy_	−0.00 ± 0.03	0.970	−0.01 ± 0.03	0.051	0.01 ± 0.02	0.131
*V*_16 Gy_	−0.00 ± 0.04	0.852	−0.02 ± 0.03	0.066	0.01 ± 0.04	0.110
*V*_14 Gy_	−0.00 ± 0.05	0.823	−0.03 ± 0.04	0.086	0.01 ± 0.05	0.328
*V*_12 Gy_	−0.02 ± 0.08	0.526	−0.03 ± 0.05	0.110	−0.01 ± 0.10	0.722
*V*_10 Gy_	−0.03 ± 0.14	0.433	−0.04 ± 0.06	0.066	−0.03 ± 0.17	0.859
*V*_8 Gy_	−0.07 ± 0.20	0.191	−0.06 ± 0.08	0.051	−0.07 ± 0.26	0.722
*V*_6 Gy_	−0.18 ± 0.35	0.009^**^	−0.10 ± 0.13	0.038^*^	−0.25 ± 0.45	0.075
*V*_4 Gy_	−0.81 ± 1.21	<0.001^**^	−0.20 ± 0.27	0.038^*^	−1.31 ± 1.44	0.003^**^
*V*_2 Gy_	−2.98 ± 4.74	0.001^**^	−0.20 ± 0.56	0.260	−5.25 ± 5.40	0.003^**^

*Note*: Statistical analysis in this study was performed using the paired Wilcoxon signed‐rank test.

*p‐Value < 0.05.

**p‐Value < 0.01.

### 3.2 CL‐VMAT versus RP versus HA

**FIGURE 1:**
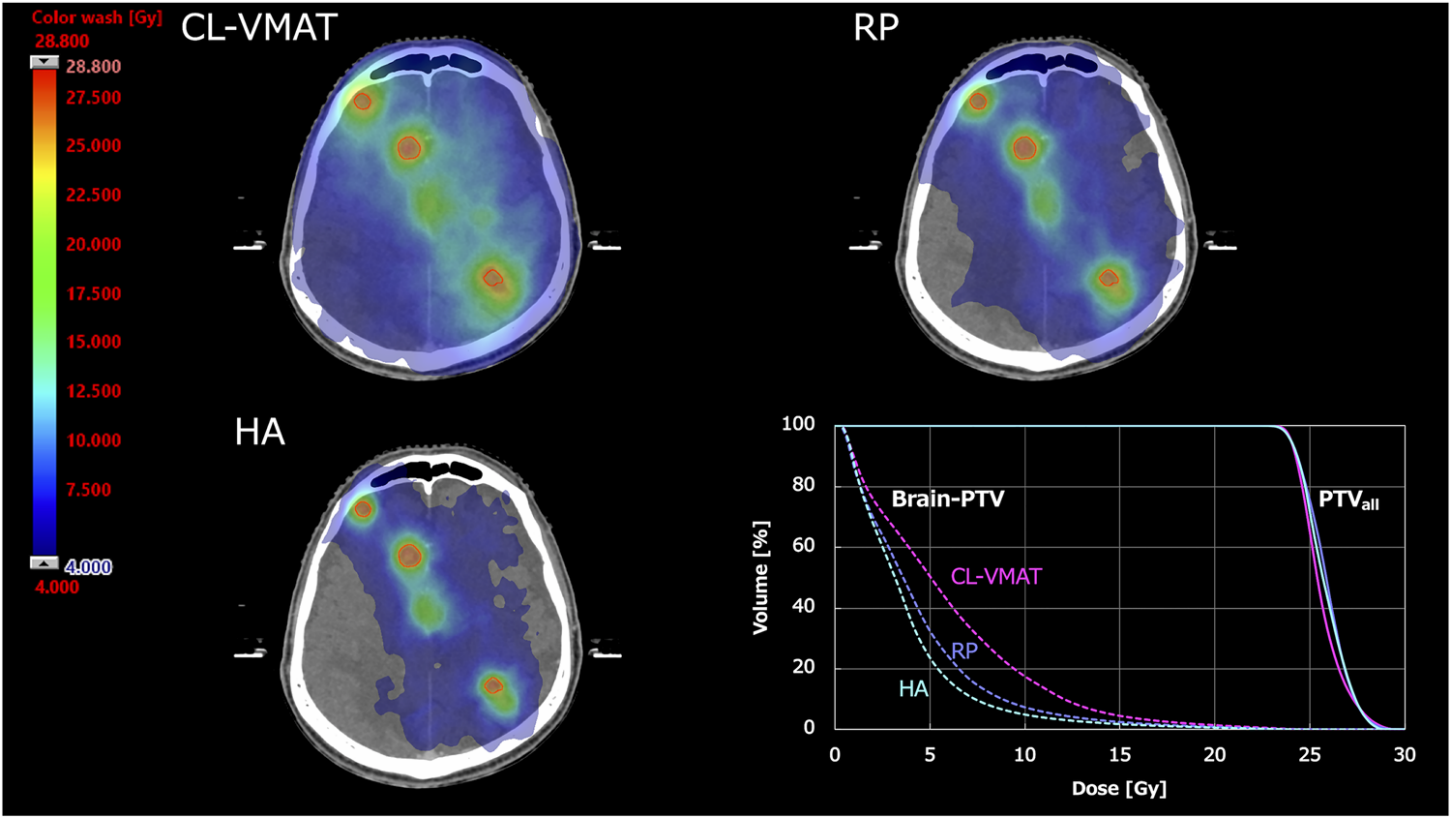
Examples of dose distributions and dose–volume histograms (DVH): Three distributions are shown as absolute dose color wash in the range from 28.8 to 4.0 Gy. The red contours represent PTV_all_.

**FIGURE 2:**
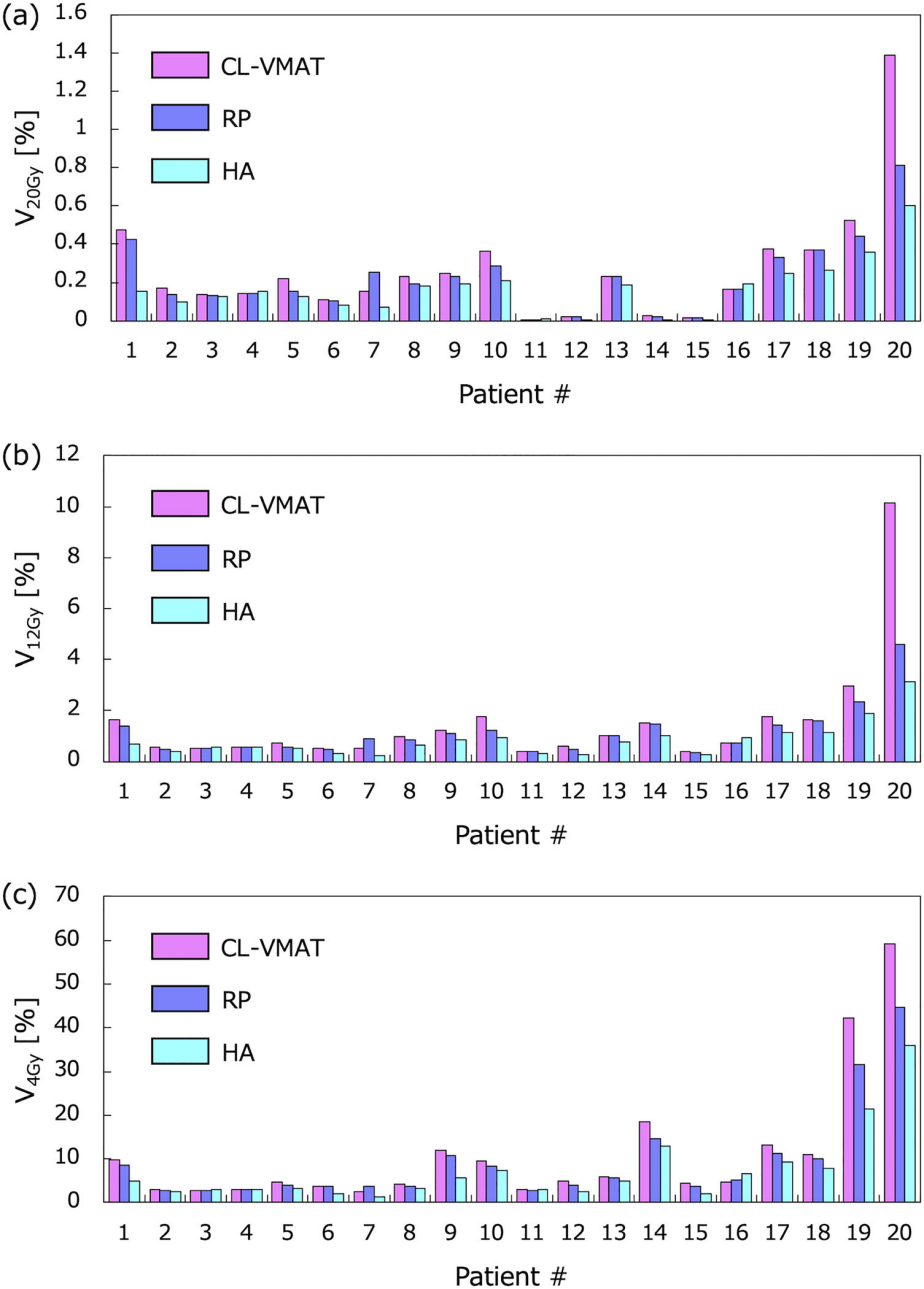
The bar graph of (a) *V*_20 Gy_, (b) *V*_12 Gy_, and (c) *V*_4 Gy_ for Brain − planning target volume (PTV) in three plans (pink, purple, and sky blue for clinical volumetric modulated arc therapy [CL‐VMAT], RP, and HA), respectively: Patients 1–8 had a single metastasis, and patients 9–13 had 2 metastases, whereas patients 14 and 15 had 3, patients 16 and 17 had 4, patient 18 had 6, patient 19 had 8, and patient 20 had 10 metastases.

**FIGURE 3:**
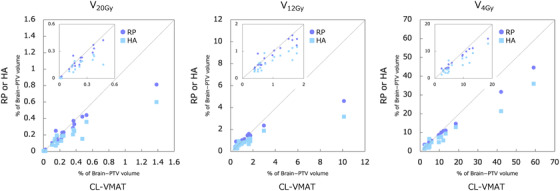
The scatter plots of *V*_20 Gy_, *V*_12 Gy_, and *V*_4 Gy_ (%) for Brain − planning target volume (PTV) in clinical volumetric modulated arc therapy (CL‐VMAT), RP, and HA: The detail plots in low value range are shown in each figure.

In CL‐VMAT, the mean ± SD of PTV volume, *V*_10 Gy_, *V*_12 Gy_, and *V*_14 Gy_ for Brain − GTV were 4.72 ± 2.81 (range, 1.1–13.7 cm^3^), 32.20 ± 44.67, 22.55 ± 34.36, and 14.62 ± 19.34 cm^3^, respectively. Figure 1 shows that CL‐VMAT had the largest volume exposed to low dose in the three plans. In 16 out of 20 patients, CL‐VMAT provided the highest *V*_12 Gy_, whereas HA provided the lowest (Figure 2). Figure 3 shows the scatter plots of *V*_20 Gy_, *V*_12 Gy_, and *V*_4 Gy_ for Brain − PTV in the three plans. RP provided significantly lower brain dose than CL‐VMAT (mean ± SD of *V*_20 Gy_: 0.22 ± 0.19 [RP] vs. 0.27 ± 0.30 [CL‐VMAT], *p* = 0.007, *V*_12 Gy_: 1.13 ± 0.94 vs. 1.52 ± 2.08, *p* = 0.002, and *V*_4 Gy_: 9.24 ± 10.40 vs. 11.14 ± 14.07, *p* = 0.002). HA also provided significantly lower brain dose than CL‐VMAT (mean ± SD of *V*_20 Gy_: 0.16 ± 0.14, *p* = 0.001, *V*_12 Gy_: 0.83 ± 0.66, *p* < 0.001, and *V*_4 Gy_: 7.16 ± 8.04, *p* = 0.001).

As shown in Table 4, differences in *D*_98%_, *D*_80%_, *D*_50%_, and *D*_20%_ of PTV_all_ between CL‐VMAT and RP or HA were close to zero. Comparisons between the three plans were made between the same patients, that is, the same prescription doses, but in Table 4, the averages were calculated for the plans with different prescriptions as well. The maximum absolute differences of *D*_98%_, *D*_80%_, *D*_50%_, and *D*_20%_ were 0.16, 0.30, 0.47, and 0.57 Gy for RP (RP minus CL‐VMAT), and 0.35, 0.16, 0.30, and 0.37 Gy for HA (HA minus CL‐VMAT), respectively. The CI were also comparable between CL‐VMAT and RP (*p* = 0.179) or HA (*p* = 0.794). *D*_max_ to the OARs (brainstem, chiasm, and both optic nerves) were comparable with no significant difference (*p* > 0.05) between CL‐VMAT and other plans.

**TABLE 4:** Parameters for PTV_all_ and organ at risks compared with clinical volumetric modulated arc therapy (CL‐VMAT)

Structure	Parameter	CL‐VMAT	RP		HA	
		Mean ± SD	Mean ± SD	*p‐*Value	Mean ± SD	*p‐*Value
PTV_all_	*D*_98%_ (Gy)	23.04 ± 1.42	23.04 ± 1.41	0.575	23.09 ± 1.50	0.232
	*D*_80%_ (Gy)	24.28 ± 1.55	24.35 ± 1.58	0.037^*^	24.21 ± 1.56	0.067
	*D*_50%_ (Gy)	25.24 ± 1.69	25.41 ± 1.75	<0.001^**^	25.12 ± 1.70	0.037^*^
	*D*_20%_ (Gy)	26.02 ± 1.91	26.23 ± 1.94	<0.001^**^	25.95 ± 1.91	0.351
	CI	0.89 ± 0.05	0.89 ± 0.07	0.179	0.89 ± 0.05	0.794
Brainstem	*D*_max_ (Gy)	3.74 ± 4.00	3.85 ± 4.18	0.411	3.73 ± 3.83	0.601
Chiasm	*D*_max_ (Gy)	1.45 ± 1.26	1.51 ± 1.37	0.748	1.49 ± 1.21	0.601
Optic nerve (L)	*D*_max_ (Gy)	1.35 ± 1.56	1.37 ± 1.56	0.763	1.32 ± 1.61	0.872
Optic nerve (R)	*D*_max_ (Gy)	1.32 ± 1.40	1.33 ± 1.43	0.717	1.31 ± 1.43	0.778

Abbreviations: CI, conformity index; HA, VMAT plans reoptimized in HyperArc system; PTV_all_, a union of all planning target volumes; RP, VMAT plans reoptimized in RapidPlan system with Model‐HA.

*p‐Value < 0.05.

**p‐Value < 0.01.

**FIGURE 4:**
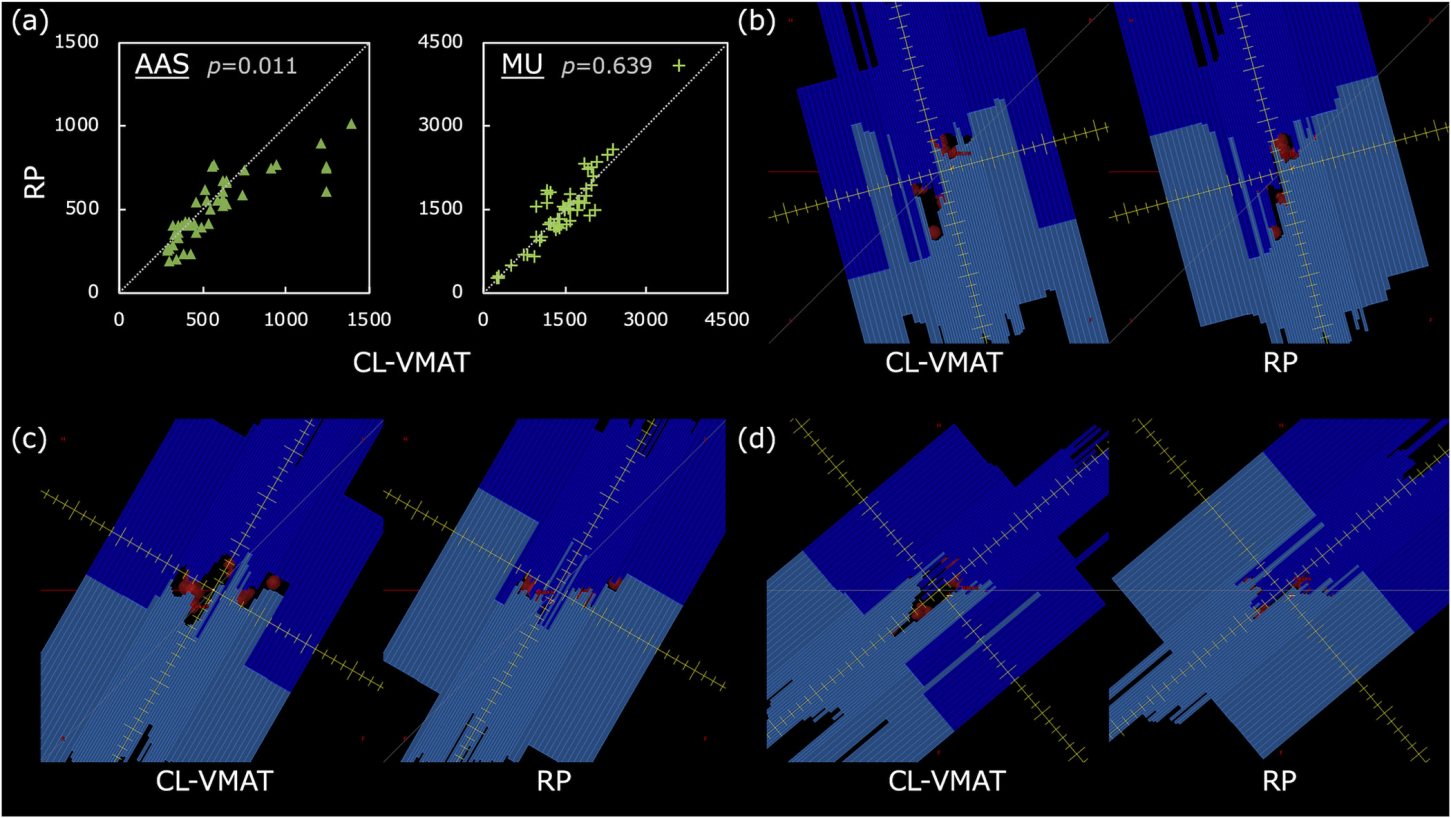
The scatter plots of (a) area aperture size (AAS) and monitor units (MU) and examples of (b–d) the beams eye views pairs of clinical volumetric modulated arc therapy (CL‐VMAT) and RP for 12 metastases case: The red structures in views represent PTV_all_.

Figure 4a shows that CL‐VMAT provided significantly larger AAS (*p* = 0.011) than RP, but comparable MU (*p* = 0.639). The mean values (±1 SD) of AAS and total MU at each field were 570.5 ± 274.0, 1481.9 ± 578.4 MU for CL‐VMAT, and 505.9 ± 188.9, 1499.8 ± 651.7 MU for RP. Although the AAS of RP was significantly lower than that of CL‐VMAT, the mean gamma pass rate of RP 20 plans (95 fields) was 99.8% (median, 100%; range, 94.8%–100%), a clinically useful level. Some examples of beam eye views (BEV) shown in Figure 4b–d indicate that the BEV of CL‐VMAT were larger than those of RP.

## 4 DISCUSSION

This study presented a RapidPlan model trained with HyperArc plans and evaluated the dosimetric potential of the model. We were able to create a model trained on HyperArc. With the same beam settings as HyperArc, the model‐based plan was able to reduce the brain dose as much as HyperArc. However, HA reduced the brain dose even more than RP, indicating that the dose distribution is highly dependent on the beam placement as well as the model performance. As both KBP and HyperArc are effective methods for reducing normal brain dose in multiple brain metastases treatments, our comparison of their effectiveness was of great value in helping to determine the planning approach.

Previous studies concluded that RapidPlan provided significant improvement in OAR sparing with similar coverage for targets and remaining OARs in prostate, rectum, lung, esophagus, and head‐and‐neck cancers.^13^, ^14^, ^15^, ^16^, ^17^, ^18^ Results in this study showed that Model‐HA provided significantly lower irradiated volume of Brain − PTV in the range of 20–4 Gy than CL‐VMAT, while providing a comparable dose to other critical OARs and similar target coverage. This shows the effectiveness of RapidPlan in planning for brain metastases. Considering physical characteristics of the plans, this dose reduction in normal brain could be attributed to the significantly smaller AAS of the RP. Blonigen et al. reported that volume in the range of *V*_8 Gy_ to *V*_16 Gy_ was the best predictor of brain radionecrosis^27^; given this evidence, brain dose reduction using RapidPlan is clinically meaningful.

The results of the model validation indicated that the dose reduction capability of the Model‐HA was comparable to that of HyperArc system, but the comparison of RP and HA using the clinical CL‐VMAT showed that HA produced a better dose distribution. Therefore, this suggests that the dose distribution is greatly affected by the arc geometry and collimator angle setting as well as the model performance. As shown in Table 2, RapidPlan has high optimization potential but requires manual geometry setting. On the other hand, HyperArc has high optimization potential and can even automatically optimize the geometry. These allowed HA to achieve a lower brain dose than RP with the same arc geometry and collimator angle as CL‐VMAT. The RapidPlan model could help to improve the plan quality of CL‐VMAT, but in addition, setting the proper arc geometry and collimator angle would also be effective.

High‐quality plans should be selected for the training data of KBP model. Ohira et al. compared HyperArc with conventional VMAT and demonstrated the superiority of HyperArc on dose conformity, rapid dose falloff, and sparing dose for normal brain tissue.^11^ Vergalasova et al. reported that HyperArc provided similar or better low‐dose spread than Gamma Knife, while maintaining excellent conformity as well as minimizing inter‐planer variability and beam‐on time.^23^ Taken together, these reports suggest that HyperArc is one of the most effective approaches for the radiotherapy of brain metastases. Therefore, it would be an appropriate and brand‐new training data for KBP models. Kishi et al. showed that the RapidPlan model in the single‐isocenter VMAT for multiple brain metastases was equivalent in dose distribution to the clinical plan with a single optimization.^21^ The fact that Model‐HA was able to improve the dose distribution of CL‐VMAT may be due in part to the high plan quality used for training. A significant advantage of using RapidPlan models is the capacity to share models, as shown by Schubert et al. who reported on model exchange among different clinical institutes joined in a cooperative framework.^28^ If the benefits of HyperArc's powerful OAR dose sparing capability can be received at multiple institutes by using KBP technology, it will be of great clinical value in terms of improving the radiotherapy quality for brain metastases cases.

Some limitations in this study should be considered. First, the data used to create, validate, and test the Model‐HA were obtained from a single institution. In order to assess the applicability of our model, it is necessary to validate it using clinical data from other institutions. If there are differences in contouring methods or treatment planning policies among facilities that share models, the models may not estimate DVH well. Before using the model, users should compare it to their facility's original treatment plan to ensure that the plan quality will improve and that the model will properly produce a distribution consistent with their facility's policies. Second, because of the need to collect a large number of cases, the CL‐VMAT plans used in this study included variations in energy and prescribed dose. As we only compared plans among the same patients, it would not directly affect the comparison, but it would be preferable to examine the energy and total dose dependence of dose sparing as well. Third, when creating RP and HA, *D*_50%_ and *D*_20%_ for targets were significantly increased compared to the clinical plan; it was extremely difficult to make the DVH of PTV exactly the same as that of CL‐VMAT. It was possible that changes in the isodose line of the dose distribution affected the dose falloff.

## 5 CONCLUSION

This study generated RapidPlan model trained with HyperArc and evaluate its dosimetric performance. The Model‐HA had the potential to significantly improve the plan quality of conventional VMAT plans. The arc geometry and collimator angle selection were also important factors in improving the dose distribution. Sharing the model would allow facilities that do not have a license to use HyperArc to improve their plans.

## AUTHOR CONTRIBUTIONS

All authors (1) made substantial contributions to the study concept or the data analysis or interpretation, (2) drafted the manuscript or revised it critically for important intellectual content, (3) approved the final version of the manuscript to be published, and (4) agreed to be accountable for all aspects of the work.

## CONFLICT OF INTEREST

The authors declare that there are no conflicts of interest.
